# Efficient translation of Eggplant mottled dwarf nucleorhabdovirus N and X genes requires both 5′ and 3′ UTRs

**DOI:** 10.1186/s12985-021-01601-4

**Published:** 2021-06-26

**Authors:** Ghobad Babaei, Amir Massah, Mina Koohi Habibi

**Affiliations:** 1 Plant Protection Research Department, Chaharmahal and Bakhtiari Agricultural and Natural Resources Research and Education Center, AREEO, Shahrekord, Iran; 2 Department of Plant Protection, College of Agriculture, Isfahan University of Technology, Isfahan, Iran; 3 Department of Plant Protection, Faculty of Agricultural Sciences and Engineering, University College of Agriculture and Natural Resources, University of Tehran, Karaj, Iran

**Keywords:** Eggplant mottled dwarf virus, Rhabdovirus, Translation

## Abstract

**Background:**

Circularization of RNA mediated by association of translation factors and RNA elements in 5′ and 3′ UTRs is a common feature for translation control in eukaryotes. There is no information about translation in plant rhabdoviruses and little information is known in animal rhabdoviruses.

**Methods:**

The role of 5′ and 3′ UTRs in two genes of EMDV in translation were studied using luciferase constructs and RNA structures of these sequences were analyzed by SHAPE and Inline probing.

**Results:**

We have found that efficient translation of N and X mRNAs of nucleorhabdovirus Eggplant mottled dwarf virus (EMDV) requires elements present in both 5′ and 3′ UTRs. Luciferase reporter constructs containing precise 5′ and 3′ UTRs of the N and X genes had substantially higher translational activity compared with constructs containing only the 5′ or 3′ UTR. The 3′UTR of *carmovirus* Turnip crinkle virus, which contains a well-characterized cap-independent translation enhancer, was unable to complement the lack of EMDV 3′ UTR. Addition of cap analog to luciferase constructs containing the UTRs of the N gene did not restore translation, and translation of the reporter construct in the absence of the 5′ cap was higher than the capped construct. No RNA-RNA interactions between 5′ and 3′ UTRs were detected by EMSA or in-line cleavage structural assays. Deletion of 11 nucleotides from the 3′ terminus negated the synergistic activity of the 3′UTR.

**Conclusions:**

The results with RNA-RNA interaction suggesting that translational synergy between the UTRs may utilize alternative means. Mutation analysis in 3′UTR suggesting that the polyadenylation signal sequence contained in this location may play a critical role in translation.

## Background

The Rhabdoviridae family, in the order of Mononegavirales, contains viruses with negative-sense RNA genomes that include plant-infecting and animal-infecting viruses. Rhabdovirus genes contain the following five canonical genes (beginning at the 5′ end of the complementary plus-strand) that encode: nucleoprotein (N), phosphoprotein (P), matrix protein (M), glycoprotein (G) and large protein (L). The conservation of genes and gene order among all rhabdoviruses suggests that these viruses likely share common mechanisms of replication, transcription and translation. However, the low sequence similarity among orthologues of these proteins suggests that considerable host adaptation has occurred [1–4]. mRNAs encoding the individual proteins are generated by the L-gene-encoded RNA-dependent RNA polymerase using a well-characterized start-stop mechanism that results in high levels of the 3′ proximal gene’s transcripts (N), and step-wise 30% reductions in the levels of downstream transcription products [5, 6]. Unlike transcription, little is known about rhabdoviruses translation. Although rhabdovirus mRNAs are similar to host mRNAs in containing a 5′ m7GpppN cap and a 3′ poly(A) tail, translation of these mRNA appears to require a mechanism distinct from the host (6). Host translation initiation is a complex process that makes use of the 5′ m7GpppN cap and 3′ poly(A) tail and numerous eukaryotic initiation factors (eIFs) including the cap-binding protein eIF4E to recruit the 40S ribosomal subunit and associated ternary complex (met-tRNAmet, eIF2 and GTP) to the mRNA 5′ end [7]. eIF4E is a subunit of the eukaryotic translation initiation factor complex eIF4F, which also includes eIF4G, a core scaffolding protein. By simultaneously interacting with eIF4E and 3′ terminal-bound poly (A)-binding protein, eIF4G acts as a bridge between the cap and poly(A) tail, which is thought to facilitate translation reinitiation by post-termination ribosomes [8, 9]. The 43S ribosome preinitiation complex is recruited to the 5′ end of the mRNA and then direction-ally transits along the template in the 3′ direction until recognizing an initiation codon in the proper context. The 60S ribosomal subunit then joins the preinitiation complex, followed by translation initiation [10, 11].

Inhibition of canonical cap-dependent translation by depletion of translation factors eIF4E and eIF4G or rapamycin treatment had no effect on translation of the Vesicular stomatitis virus (VSV), suggesting that rhabdoviruses are translated by a cap-independent mechanism [12, 13]. VSV inhibits host translation during infection by dephosphorylating 4E-BP1, a protein that binds to eIF4E [12, 14, 15] and mutations in loop structures of the M protein reduce translation, suggesting that M may substitute for the eIF4F complex [16]. In addition, binding of cellular poly(C) binding protein 2 to the 3′ UTR of G mRNA plays a positive role in translation by enhancing mRNA stability [17] which may be necessary to overcome lower transcription levels due to start-stop transcription and its location in the genome [18].

**Fig. 1:**
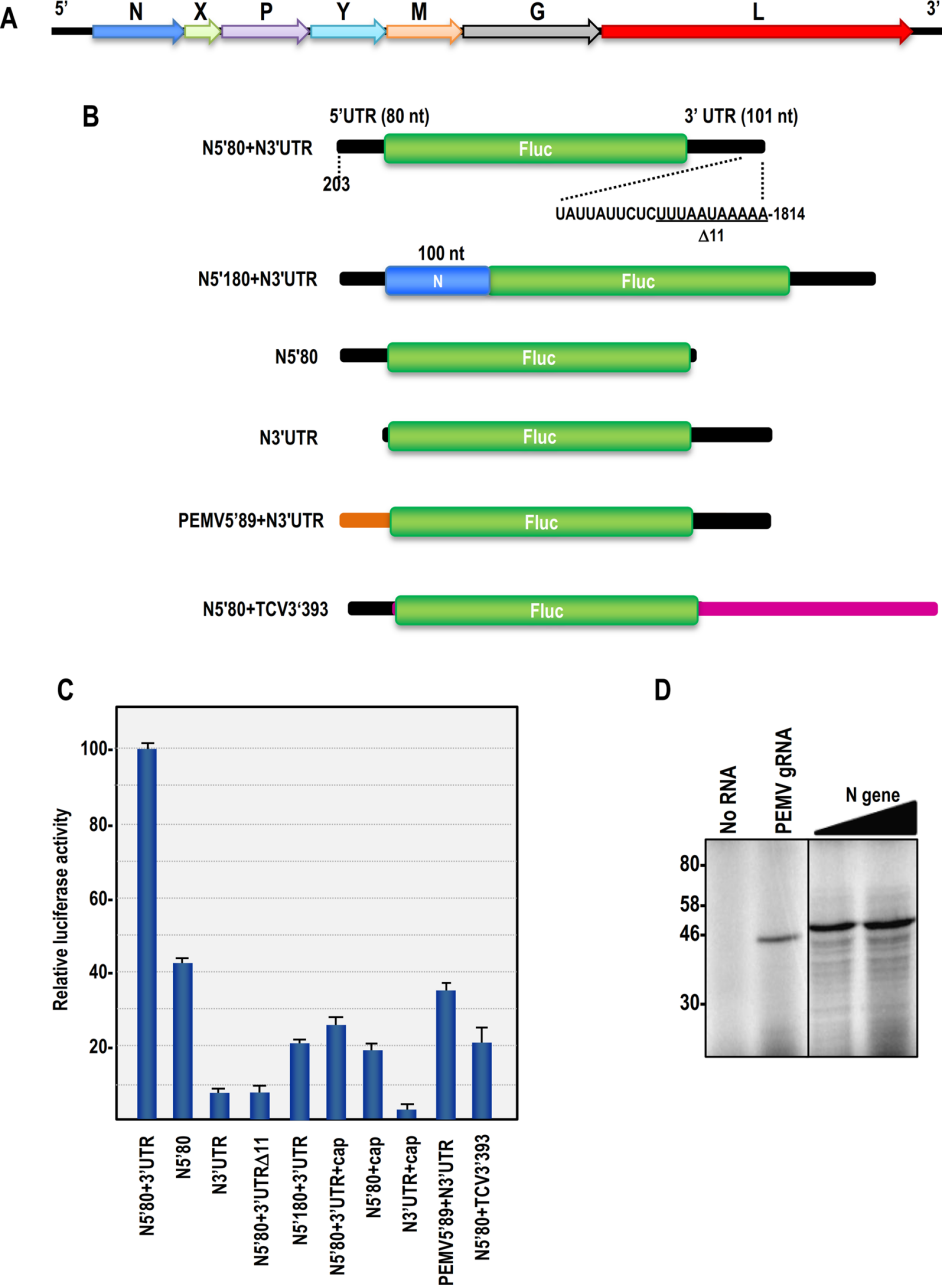
**A** Schematic representation genomic organization of EMDV containing 7 ORF (+ strand of virus genome). **B** Schematic representation of Luciferase constructs for N gene of EMDV. N gene constructs contain 80 and 180 nt of 5′ N gene in 5′ side and 3′UTR of N gene in 3′ side of the luciferase gene, and luciferase construct contains precise 80 nt of 5′ N gene in 5′ side and 3′ UTR of TCV in 3′ side of the luciferase gene. Schematic representation of luciferase construct contains N UTRs and Predicted structure for 3′ UTR of N gene which selected region of 11 nt in mutagenesis has been shown. **C** Relative In vitro translation activity in luciferase constructs. Luciferase activity in luciferase reporter transcript containing the 5′ and 3′ fragments from N gene include construct contain TCV (393) 3′UTR capped construct and mutants (mutant of the N5′80-fluc-N3′UTR construct with 11 nt (position 1804–1814) deletion from the end of 3′ UTR). **D** In vitro translation of EMDV N gene with different amount of RNA in wheat germ extract. Lane 1, control without any RNA Lane 2, PEMV2′s RNA as control for translation system. Lane 3 and lane 4, 0.5 μl and, 2 μl of N gene RNA

Plant-infecting rhabdoviruses are now 6 genera of unsegmented and segmented genomes that nucleorhabdoviruses composed of viruses that replicate in the nucleus [4, 19, 20]. Eggplant mottled dwarf virus (EMDV), a member of the Alphanucleo-rhabdoviruses, has a broad host range including solanaceous crops (potato, tomato, eggplant and tobacco), cucurbit, muskmelon and some weeds, and is transmitted by the leaf-hopper Agallia vorobjevi [21, 22]. EMDV has an unsegmented RNA genome of 13,154 nt with a genome organization similar to that of the type member Potato yellow dwarf virus (PYDV) [23]. From the 5 end of the (+)-strand, EMDV ORFs encode N, X (a protein of unknown function), P, Y (the plant-specific putative movement protein), M, G, and L (Fig. 1a) [24]. Nothing is known about the translation of EMDV or any plant-infecting rhabdoviruses. Here we describe the importance of the 5′ and 3′ UTRs for translation of EMDV N and X gene reporter constructs along with their unusual structural features.

## Methods

### Construction of luciferase reporter constructs

**Fig. 2:**
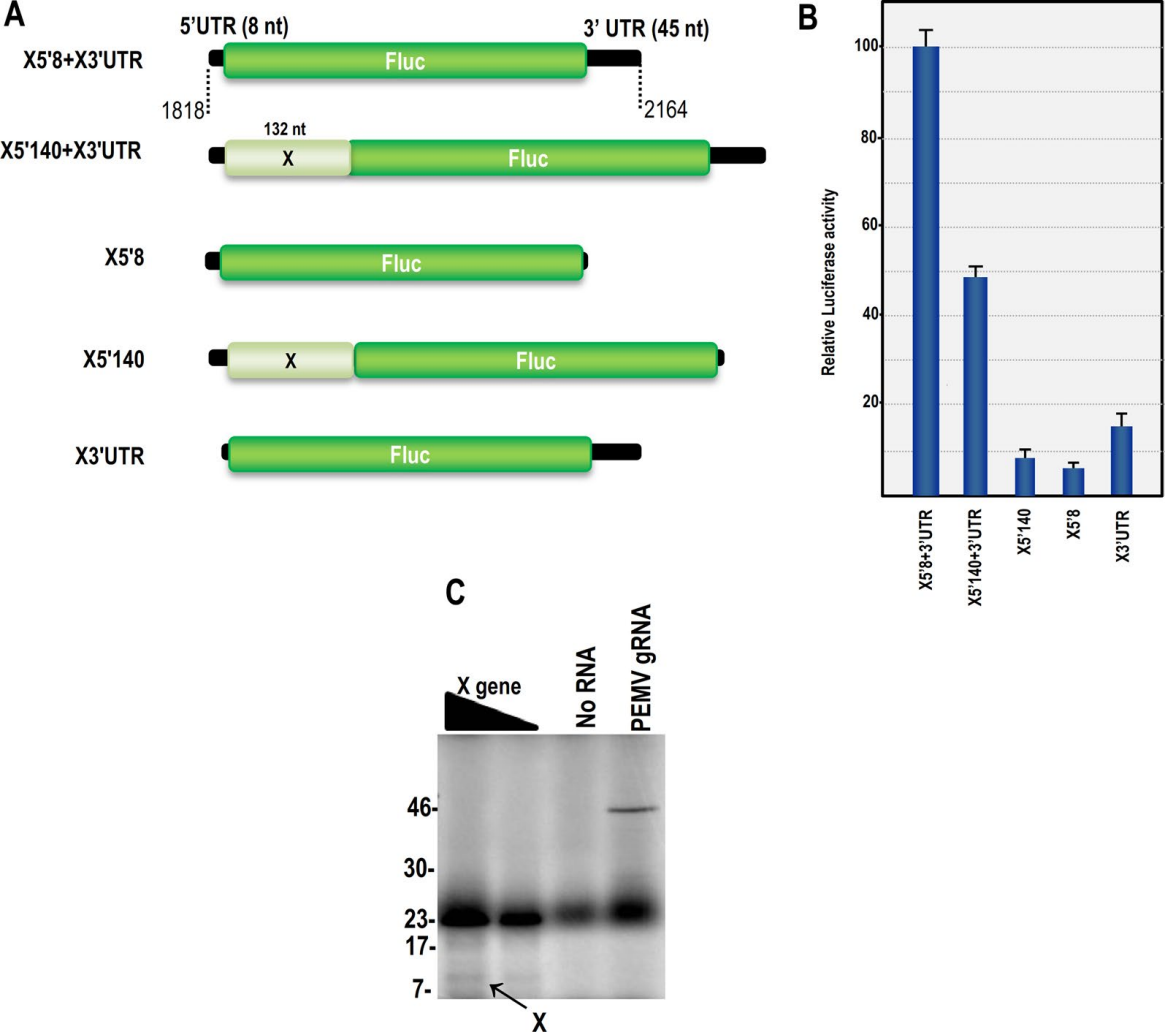
**A** Schematic representation Luciferase constructs for X gene of EMDV. X gene constructs contain 8 and 140 nt from 5′ X gene and 3′ UTR of X gene in 5′ and 3′ sides of the luciferase gene. **B** Luciferase activity in luciferase reporter transcript containing the 5′ and 3′ fragments from X gene. **C** In vitro translation of EMDV X gene in wheat germ extract with different amount of RNA. Lane 1, 2 μl and lane 2, 0.5 μl of RNA. Lane 3, control without any RNA Lane 4, PEMV’s RNA as control

For the N gene UTRs, firefly luciferase (Fluc) reporter constructs contained the precise 3′ UTR (101 nt) and either the 5′ UTR (80 nt) or the 5′ UTR with 100 nt of downstream coding sequence (Fig. 1b). The X gene constructs contained the 3′ UTR (45 nt) and either the 5′ UTR (8 nt) or the 5′ UTR with 132 downstream nucleotides including polyadenylation signal at end of 3′ UTR (Fig. 2a). The N 3′ UTR was also replaced with the 3′ terminal 393 nt of TCV, which includes the complete TCV 3′UTR, in constructs that also contained the precise 5′ UTR of the N gene (Fig. 1b). All constructs contained a T7 RNA polymerase promoter followed by BamHI and SacI restriction sites and all 5′ fragments derived from N and X mRNAs were inserted between the T7 promoter and luciferase start codon using these sites. In all constructs, 18 additional nt (including the 6 nt restriction site) were present between the T7 promoter sequence and the 5′ UTRs. The 3′ UTRs were inserted adjacent to the stop codon of the Fluc coding region using *Ssp*I and *Nru*I restriction sites. 45 additional nucleotides (including the 6 nt restriction site) were present between the Fluc stop codon and the 3′ UTR. All fragments were generated by PCR using primers designed for digestion with *BamH*I/*Sac*I sites for the 5′ fragments and *Nru*I/*Ssp*I for 3′ fragments and then inserted into the appropriate vector sites. Reporter constructs were linearized with *Pml*I and *SspI* before transcription using T7 RNA polymerase to generate both capped and uncapped RNAs according to the manufacturer's instruction. Deletion of 11 nt from the 3′ terminus of 5′N80-Fluc-N3′UTR was generated using oligonucleotide-mediated site-directed mutagenesis [25]. All constructs were verified by sequencing.

### In vitro translation

Transcripts synthesized in vitro were purified following excision from 1% agarose gels. Cap analog (m7G(5) ppp(5) G) was added to selected constructs according to the manufacturer’s protocol (Ambion). Unincorporated cap was removed from synthesized transcripts using a G25 column (GE) followed by fragment purification through a 2% agarose gel and RNA band excision. Purified RNA normalized and equal molar amount (0.1 pmol) of them were added into the in vitro reaction using wheat germ extracts (WGE; Promega). After 1 h incubation at 25 °C, luciferase activity was measured using a luciferase assay reporter system (Promega) in a Modulus microplate multimode reader (Turner Bio-Systems). Transcripts of EMDV N and X mRNAs were also translated in vitro in WGE using [35S] methionine and an amino acid mixture in the absence of unlabeled methionine. Transcripts were synthesized at 37 °C for 2.5 h and then subjected to phenol–chloroform extraction and ethanol precipitation. RNA integrity was verified on 1% agarose gels. Products were subjected to electrophoresis through 5% polyacrylamide gels.

### In-line RNA structure probing

Fragments containing the N gene 5′ UTR (80 nt, positions 203–282) or 3′ UTR (101 nt, positions 1714–1814) were amplified by PCR using a primer containing a T7 promoter sequence at the 5′ end, followed by synthesis of transcripts using T7 RNA polymerase. In-line probing was performed as previously described [26–28]. 5′ UTR and 3′ UTR N gene transcripts were purified from agarose gels and dephosphorylated using Antarctic phosphatase (NEB). The 5′ end was labeled with [ɣ-32P] ATP and polynucleotide kinase and purified through 5% polyacrylamide gels. Labeled fragments were denatured by heating at 75 °C and then slowly cooling to room temperature. In-line cleavages took place at 25 °C for 14 h. RNA cleavage ladders were generated by incubating 5 pmol of 5′-labeled RNA using 1 μg of yeast tRNA in in-line cleavage buffer (50 mM NaHCO3/Na2CO3, pH 9.2; 1 mM EDTA, pH 8.0) for 5 min at 95 °C. The RNase T1 ladder that denotes the position of guanylates was generated by incubating 5 pmol of 5′-labeled RNA with 2 μg of yeast tRNA, 20 mM sodium citrate pH 5, 1 mM EDTA, 7 M urea and 1-unit RNase T1 (Ambion) for 5 min at 25 °C. Reactions were phenol extracted, ethanol precipitated, and the RNA pellet resuspended in gel loading buffer II (Ambion). The sample was heated for 2 min at 95 °C, snap cooled on ice and subjected to electrophoresis through 8% polyacrylamide gels containing 8.0 M urea followed by autoradiography.

To investigate if two RNA fragments were interacting thereby reducing in-line cleavage at interacting residues, tenfold excess unlabeled 3′ or 5′ UTR N-gene fragments were combined with the labeled fragment and cleavages allowed to take place as described above.

### Selective 2′ Hydroxyl Acylation analyzed by Primer Extension (SHAPE) analysis of N gene 5′ and 3′ UTRs

SHAPE was carried out on a full-length EMDV N gene as described previously [29, 30]. Briefly, PCR amplification was used to generate N gene cDNA with a T7 promoter sequence introduced at the 5′ end. Full-length N transcripts (6 pmols) were denatured by heating at 65 °C for 5 min and kept on ice for 3 min. SHAPE folding buffer [80 mM Tris–HCl pH 8, 11 mM Mg (CH3COO)2 and 160 mM NH4Cl] was added to the transcripts and the mixture was incubated at 37 °C for 30 min. N transcript RNA was split into two reaction tubes with N-methyl isatoic anhydride (NMIA) added to one tube (final concentration 15 mM in a final volume of 24 μl) and DMSO was added to the second tube as a control. Samples were then incubated for 45 min at 37 °C. Following ethanol precipitation, sample pellets were resuspended in 30 μl of 0.5 M Tris–EDTA buffer (10 mM Tris pH 8, 1 mM EDTA). Reverse transcription was carried out using 0.5 μl (20 unit) SuperScript III reverse transcriptase (Invitrogen) and [ɣ-32P] end-labeled oligonucleotides complementary to sequences that were 60 nt, 100 nt and 200 nt downstream of N gene 3′ end belong to the downstream sequences, were used for primer extension. Four picomoles of RNA were mixed with 6 pmol of labeled oligonucleotide in a total volume of 34 ul, incubated at 65 °C for 5 min, then 55 °C for 2 min, and 25 °C for 5 min. Four microliters of 5X Superscript III buffer and nucleotides (100 mM DTT, 10 mM dNTP mix) were added along with 20 U of Superscript III enzyme and 2 μl water and the reaction heated at 52 °C for 1 h. Radioactively labeled extension products were subjected to 8% denaturing polyacrylamide gel electrophoresis and exposed to autoradiography.

### Electromobility shift assays (EMSA)

EMSA was performed based on a previously described procedure [31]. EMDV N gene 5′ and 3′ UTR fragments were labeled with [ɣ-32P] ATP. Labeled and unlabeled fragments were heated to 75 °C and cooled down to room temperature. Labeled RNAs (2 pmols) were mixed with 5 and 10 pmols of unlabeled RNA and incubated for 30 min at 25 °C. Reactions were transferred to ice and then subjected to electrophoresis on 2% agarose gels cooled to 4 °C. After electrophoresis, gel was dried and analyzed by autoradiography.

## Results

Both 5′ and 3′ UTRs are required for efficient translation of reporter constructs.

Since little is known about animal rhabdovirus translation, and nothing is known about plant rhabdovirus translation, we were interested in investigating whether sequences and/or RNA structures in rhabdovirus UTRs might help to facilitate translation of the capped genome. EMDV has a genome organization with the order 3′-N-X-P-Y-M-G-L-5′ (negative strand) (Fig. 1a), which is similar to the PYDV genome [20, 23, 24]. Based on the start-stop mechanism of transcription in rhabdoviruses, the N gene should be the most prominent transcript, and thus it was selected for this preliminary analysis. All EMDV genes, including the N gene, are flanked by 5′ and 3′ UTRs of various sizes. The EMDV N gene has a 5′ UTR of 80 nt and a 3′ UTR of 101 nt, and the 51 kDa N protein was efficiently translated in wheat germ extracts (WGE) using full-length N-gene transcripts, compared with translation of the first ORF of full-length transcripts of the genomic RNA of umbra-virus Pea enation mosaic virus (PEMV2) used as control (Fig. 1d) [24, 32].

To determine if the N gene 5′ and 3′ UTRs contain elements that promote efficient translation, luciferase reporter constructs were generated containing either the precise 5′ and 3′ UTRs (N5′80 + N3′UTR), or the individual UTRs (N5′UTR, N3′UTR) (Fig. 1b). Compared with translation of N5′80 + N3′UTR, translation of N5′UTR and N3′UTR was reduced by 2.4-fold and 14.1-fold, respectively. When the N3′UTR was combined with the 5′ 89 nt from PEMV2, translation was enhanced by fivefold compared with transcripts containing only the N 3′ UTR, suggesting that the PEMV2 5′ sequence is contributing to more efficient translation (Fig. 1c). However, translation efficiency was less than 50% of the level achieved when both 5′ and 3′ sequences originated from EMDV. Replacement of the N3′UTR with the 3′ terminal 393 nt of Turnip crinkle virus (TCV) (generating N5′80 + TCV (3933′UTR) did not improve translation over levels obtained with the N 5′UTR alone. Capping the 5′ end of N5′80 + N3′UTR, N5′UTR and N3′UTR were surprisingly inhibitory to translation possibly due to unincorporated cap, but translation ratio was the same suggesting that translation elements in the 5′ and 3′UTRs are more functional in WGE in the absence of a 5′cap, supporting previous reports that rhabdoviruses may be translated in association with possible cellular protein elements [33].

5′ and 3′ sequence comparisons in different rhabdoviruses genes and also between different genes of EMDV (like most plant rhabdoviruses) showed conservation of 5′-AACA-3′ at the 5′ end and 5′-UUUAAUAAAAA-3′ at the 3′ end of all genes. When 5′-UUUAAUAAAAA-3′ was deleted in 5′N80-Fluc-N3′UTR, translation was reduced by 12.5-fold (Fig. 1b, c). Since coding region elements near the 5′ end of plant virus genes can enhance translation [32–34], a construct was generated containing an additional 100 nt of the N gene (N5′180 + N3′UTR). The presence of this additional sequence reduced translation by fivefold, suggesting that this region is interfering with translation.

To determine if these results were applicable to other EMDV genes, translation of the X gene was also investigated. The EMDV X gene has a 5′UTR of 8 nt and a 3′ UTR of 45 nt. Transcripts synthesized corresponding to full-length EMDV X genes and translated in WGE produced an 11 kDa product (Fig. 2c). Luciferase constructs for the X gene were generated containing either the precise 5′ and 3′ UTRs (X5′8 + X3′UTR), the individual UTRs (X5′UTR, X3′UTR) or a larger sequence from the 5′ end of the X gene (X′140 + X3′UTR) (Fig. 2a). Luciferase constructs containing precise 5′ and 3′UTRs of the X gene were 16.3 and 6.3-fold more efficient at translation compared to constructs containing only the X 5′UTR and X 3′UTR, respectively (Fig. 2b). Extending the amount of X gene 5′ end sequence to 140 nt in reporter constructs containing the 3′ UTR reduced translation by 2.1-fold compared with X5′8 + X3′UTR. These results suggest that the mechanism for translation of both the N and X genes of EMDV is similar and that the presence of the 5′ and 3′ UTRs is required for efficient translation.

**Fig. 3:**
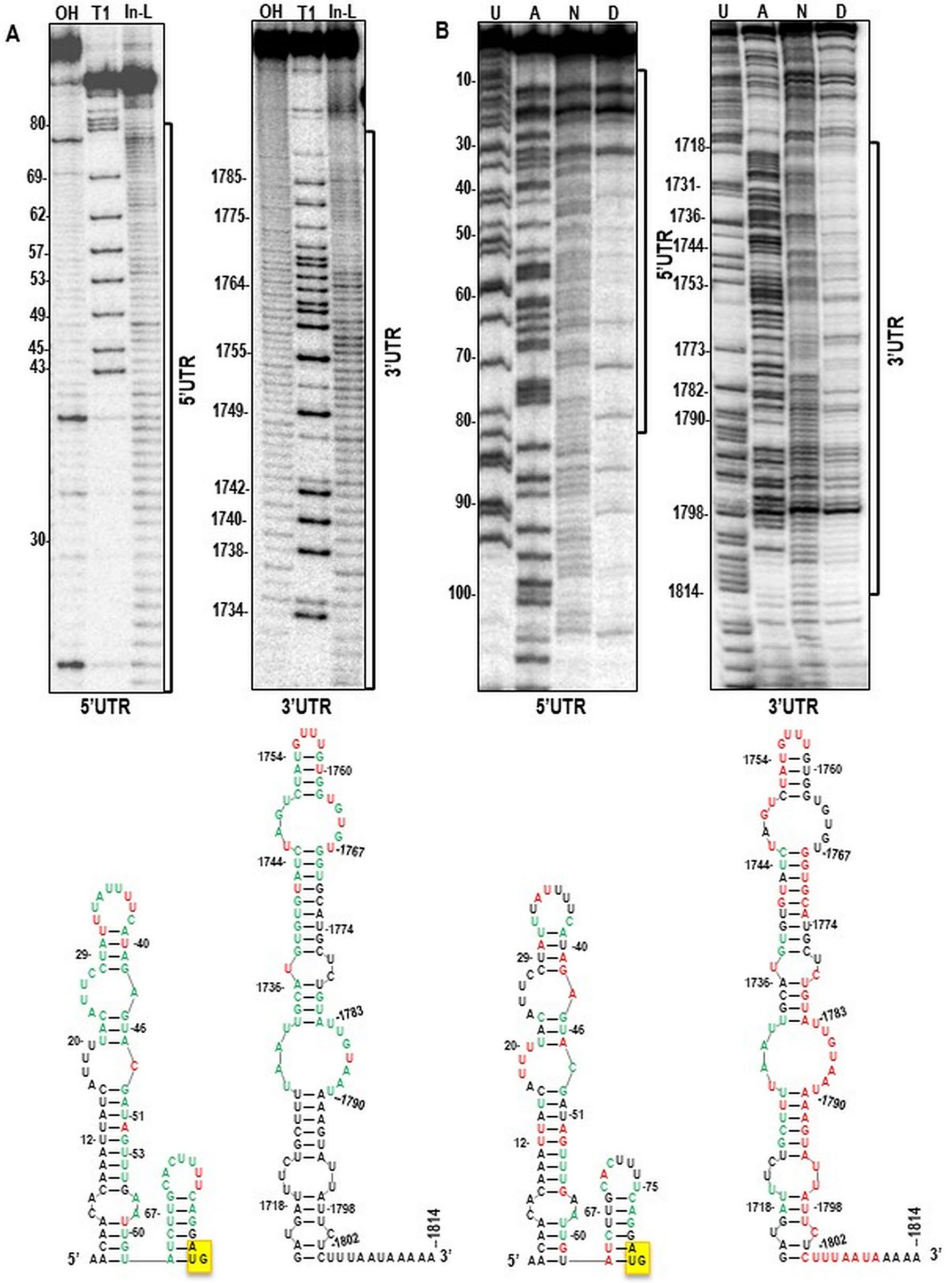
**A** RNA structure prediction by in-line probing for 5′ and 3′ UTRs of EMDV-N gene and schematic of putative structure of 5′ and 3′ UTR RNA by in-line probing. **B** SHAPE analysis of N gene UTRs and Putative structure of 5′ and 3′ UTR of EMDV-N gene using SHAPE. High reactivity/cleavage showed by red and lower reactivity/cleavage showed by green color

To determine if the 3′UTR and 5′UTR of the N and X genes harbored any structures that might be conserved and thus possibly participate in translation, we initially used the Mfold structure prediction program [35]. Structural prediction for these sequences and the 5′ and 3′ UTRs of additional EMDV and other plant rhabdoviruses did not reveal any discernible similarities outside of a short 3′ terminal single-stranded region (data not shown). To further investigate the structure of the N gene 5′ and 3′ UTRs using biochemical techniques, two methods were employed: in-line probing [27] and SHAPE (selective 2-hydroxyl acylation analyzed by primer extension) [29, 30]. In-line probing measures cleavage of the RNA backbone, which only occurs at flexible nucleotides, with the amount of cleavage correlating with the flexibility of the nucleotide. In-line probing analysis of UTRs of N gene fragments indicated that most of the residues in both regions were susceptible to cleavage and thus both fragments were significantly single-stranded and ab-sent of any extensive local secondary structure (Fig. 3a). The structure of these two regions were further investigated within the full-length N gene transcript using SHAPE, where NMIA only acylates the 2′ hydroxyl in flexible nucleotides. Although some non-reactive bases were present, both regions contained mainly reactive, flexible nucleotides (Fig. 3b). Using different primers that extended the 3′UTR into the downstream gene by 60 to 200 nt had little or no effect on the reactivity of residues in the 5′ and 3′ UTRs (data not shown). The SHAPE in positions 60, 100 and 200 nt downstream of polyA sequence in 3′ UTR or a different position in downstream of 5′ UTR end for primer extension end didn’t affect results for 3′ or 5′ UTRs. No interactions were detected between two UTRs fragments by EMSA or hot probe inline probing (data not shown)*.*

## Discussion

Translation in viruses can occur in a cap-dependent or cap-independent manner. Several plant and animal virus families with RNA genomes have internal ribosome entry site (IRES) [10, 11, 36, 37]. IRES-based alternative translation strategy can be adopted by cellular mRNA independent from cap-dependent translation [12, 33, 38, 39]. In animal viruses, IRESes are usually long (200–500 nt), structured and located in the 5′ UTRs, but in plant virus families, IRESes are shorter, less structured, and sometimes located in the 3′ UTRs [40–42]. The unstructured A/U rich sequence upstream of the CP gene in TCV and other carmoviruses has displayed IRES activity, and deletion of this region resulted in a major effect on translation [43]. Similar A/U rich sequences are conserved at the end of 3′UTR of all genes in rhabdoviruses including animal and plant rhabdoviruses [20] which has a loop structure based on computer simulated structure. Unstructured sequence regions can be used as a landing pad by ribosome to scan for the initiation codon [43]. No stable secondary structure was found in the N gene UTRs by computational and experimental analyses, indicating there are no RNA structures that affecting translation. Although the results of in vitro translation assay showed an interaction between 5′ and 3′ UTRs of N and X genes, EMSA and inline probing could not detect any interaction.

The role of host proteins in translation of rhabdoviruses mRNAs has been suggested in different studies. PCBP2 protein in interaction with the 3′UTR of G mRNA in rabies virus can help to circularization and RNA stability of viral RNA [18, 44–46]. Similar results have been seen in interaction of different host proteins and viral RNAs in translation. Subgenomic mRNA in alphavirus that lacks any structure, may be involved in the translation of viral mRNAs in association with host translation factors [33, 47].

VSV mRNA, a type member of rhabdoviridae, has a 5′ cap and 3′ poly-A tail. If, however, translation factors such as eIF4E and eIF4G are depleted or cleaved, VSV proteins synthesis prevails, suggesting that VSV has alternative translation mechanisms in association with cell proteins and translate by use of several protein like rpl40, eIf3a and eIf3d [13, 48]. Elements in viral UTRs (sequences or structure) may bind to protein factors like rpl40 directly or in association with other proteins [33]. Studying VSV protein synthesis revealed a new translation pathway of Rpl40-dependent translation for VSV mRNAs that is share as alternative cellular translation in stress response proteins [33]. VSV inhibits eIF4E translation pathway for shut-off the host defense and avoids this mechanism by using alternative translation with help of host cell proteins [48]. Ribosomal protein, Rpl40 can interact directly or indirectly with a host or viral mRNAs in association with other translation proteins like eIF3a and eIF3d and has an important role in 80S formation during translation of VSV mRNAs [48–51]. VSV mRNAs as with other Rhabdoviridae members have relatively short and unstructured 5′ UTRs that make them less sensitive than host mRNAs to alterations in the eIF4F cap-binding complex. Extending the 5′ UTR of viral mRNA to a typical length of host mRNAs has little effect on translation [15].

In the present study, we found that the 5′ and 3′ UTRs of N and X genes in EMDV are de-void of pronounced RNA secondary structure. Our results showed that 5′ and 3′ UTRs are required for efficient translation on N and X mRNAs in EMDV. It is also possible that EMDV uses protein-mediated translation when there is no detectable RNA-RNA interaction between 5′ and 3′ UTRs of N mRNA. VSV and EMDV both replicate in vector insects suggests both viruses possibly share their translation strategy. Our results of efficient translation of N and X transcript genes and also non-capped luciferase mRNA construct together with previous results for protein-mediated alternative translation in VSV, suggesting possible similar translation mechanism in plant and animal rhabdoviruses. Identification of host protein interacting with EMDV transcripts during viral infection of different hosts may provide novel insights into translation and host infection mechanisms of EMDV and other plant rhabdoviruses. Further studies are aimed to develop protein-mediated translation control in EMDV and other plant rhabdoviruses.

## Conclusions

Analysis of 5′ and 3′ UTRs sequences in plant and animal rhabdoviruses didn’t show any conserved sequence. The presence of both UTRs of n and X genes in the luciferase construct showed a higher translation level compare to one of them. Both 5′ and 3′ N gene UTR’s showed low structure in SHAPE and inline probing but didn’t show any interaction. Mutation in 11 bp of 3′UTR end decrease translation level suggests polyadenylation signal sequence in this region may play a critical role in interaction for translation. UTRs may utilize alternative ways for translation using protein factors.

## Data Availability

The analyzed information and data during the current study are available from the corresponding author on reasonable request.

## Abbreviations

UTR: Untranslated region
EMDV: Eggplant mottled dwarf virus
SHAPE: Selective 2′Hydroxyl Acylation analyzed by Primer Extension
VSV: Vesicular stomatitis virus
WGE: Wheat germ extract
NMIA: N-methyl isatoic anhydride
